# Performance of Oral Rotavirus Vaccines in Low- and Middle-Income Settings: Current Evidence, Persistent Gaps and Future Directions

**DOI:** 10.3390/v18080917

**Published:** 2026-08-21

**Authors:** Bharathikumar Sivakumar, Jason Mathiu Mwenda, Benjamin Alan Lopman, Gagandeep Kang, Tintu Varghese

**Affiliations:** 1 The Wellcome Trust Research Laboratory, Division of Gastrointestinal Sciences, Christian Medical College, Vellore 632004, India; bharathikumar2416@gmail.com; 2 WHO Regional Office for Africa (WHO/AFRO), Brazzaville P.O. Box 06, Congo; jason.mwenda23@gmail.com; 3 Department of Epidemiology, Rollins School of Public Health, Emory University, Atlanta, GA 30322, USA; benjamin.alan.lopman@emory.edu; 4 Center for Public Health, Christian Medical College, Vellore 632004, India; gkang@cmcvellore.ac.in

**Keywords:** rotavirus vaccines, LMICs, vaccine effectiveness (VE), vaccine interchangeability, GAVI-transition, long-term economic sustainability

## Abstract

Oral, live-attenuated rotavirus vaccines (ORVs) have significantly reduced severe diarrheal disease and deaths; however, vaccine performance in low- and middle-income countries (LMICs) remains lower than in high-income countries. With increasing global use, real-world data now provide important insights into strategies to improve vaccine impact in these settings. This review summarizes current evidence on the effectiveness and impact of ORVs in LMICs, with a focus on approaches to optimize performance, including alternative dosing schedules, booster doses in late infancy, and mixed vaccine regimens. Evidence on vaccine interchangeability offers practical solutions for countries facing supply disruptions and financial constraints. The review also highlights the growing importance of economic sustainability, particularly in the context of transitions from external funding support. Despite lower efficacy, ORVs continue to provide substantial public health benefits in LMICs. Sustained impact will depend on strengthening programme delivery, ensuring stable vaccine supply, and advancing next-generation vaccines. Continued evaluation of effectiveness, cost, and implementation strategies will be critical to maximizing long-term benefits in high-burden settings.

## 1. Global Burden of Rotavirus and the Public Health Value of Vaccination

Rotavirus is one of the leading causes of diarrheal deaths worldwide especially in low- and middle-income countries (LMICs) [1]. Before the widespread introduction of rotavirus vaccines, pooled pre-vaccine global estimates suggested that rotavirus caused approximately 528,000 deaths annually among children under five, with nearly half of these deaths occurring in India, Nigeria, Pakistan, and the Democratic Republic of Congo [2]. It accounted for a median of 40% of all childhood diarrheal hospitalizations [3] and was the leading cause of about 30.9 million diarrhea-related disability-adjusted-life-years (DALYs) in children under five [1]. Evidence from Bangladesh demonstrates that rotavirus illness also imposed a substantial economic burden through catastrophic household expenditure and increased healthcare costs [4]. Owing to its high burden and significant impact on child health, the World Health Organization (WHO) identified rotavirus as a priority pathogen for global vaccine development. Currently, four oral rotavirus vaccines (ORVs) are licensed for use worldwide [2,5], while additional rotavirus vaccines such as the Lanzhou lamb rotavirus vaccine in China and Rotavin-M1 in Vietnam are licensed for domestic use in the respective countries [5]. Although not WHO-prequalified and not available internationally, these regionally licensed vaccines may contribute to domestic vaccine supply and affordability. As the primary focus of this review is on globally available WHO-prequalified vaccines and strategies to optimize their use in LMICs, these regionally licensed vaccines are only briefly mentioned and are not discussed further.

The introduction of ORVs, supported in many countries by Gavi, the Vaccine Alliance, has markedly altered the global epidemiology of diarrheal disease. To date, ORVs have been introduced in more than 130 countries, and surveillance data from 49 countries have shown a mean 59% reduction in rotavirus-related hospitalizations among children under five years of age following vaccine introduction [6]. Furthermore, global analyses reported a mean 36% reduction in all-cause acute gastroenteritis mortality, showing that rotavirus vaccination has substantially prevented deaths from severe diarrheal disease in high-burden settings [6].

Despite this progress, rotavirus infection continues to cause a disproportionate burden in LMICs. The long-term impact of vaccination depends not only on vaccine efficacy, but also on biological factors, host-environment interactions, vaccine delivery strategies, and the strength of the national immunization programs. In many LMICs, programme sustainability is also affected by economic pressures, including the gradual withdrawal of Gavi support, limited public health budgets, and broader geopolitical and health system challenges. These factors highlight the need to examine rotavirus control beyond vaccine performance alone. This review therefore synthesizes available evidence on the effectiveness of ORVs in LMICs and explores the biological, programmatic, and economic determinants of their impact. It further identifies key challenges to long-term sustainability and outlines priorities to strengthen rotavirus control in high-burden settings.

## 2. Real World Effectiveness of Oral Rotavirus Vaccines in LMICs

Evidence from post-licensure studies across diverse LMIC settings (Table 1) shows that the effectiveness of ORVs generally ranges from 40% to 70%, with most estimates clustering between 50% and 60%, broadly consistent with the vaccine efficacy observed in randomized clinical trials conducted in similar settings [5,7,8]. Also, the effectiveness pattern observed is consistent across WHO-prequalified vaccines, with the newer vaccines, Rotavac and Rotasiil, providing protection comparable to Rotarix and RotaTeq, although data for the former remain more limited in scale and geographic coverage [5,8,9,10]. Overall, the vaccine effectiveness (VE) is notably lower than that reported from high-income settings (85–98%) [11,12,13]. Collectively, the evidence suggests that reduced ORV effectiveness is likely driven by multiple biological and environmental factors that are poorly understood which is also documented in the underperformance of other live oral vaccines, including oral polio (OPV) and oral cholera vaccines [14,15].

ORV performance in LMICs tends to decline with age, with protection being generally highest during the first year of life but may decrease during the second year of life, particularly in high-burden settings [5,7,12]. Although some studies have shown sustained protection into the second year of life [16,17,18,19,20,21,22,23], waning of immunity appears to be more common in LMICs, whereas in low-burden settings protection is often maintained up to 3–4 years of age. Early repeated rotavirus can partially explain the lower VE observed in the second or later years of life [24,25]. In high-incidence settings, unvaccinated children acquire natural immunity quickly, which narrows the vaccinated-unvaccinated contrast over time and can make VE appear to wane even when some vaccine protection persists. However, findings indicate that the greatest public health benefit of vaccination in high-burden settings is achieved in early infancy, with even partial protection causing reductions in hospital admissions, severe disease, and deaths [9,10,11,13]. Consistent with this, global surveillance data show that the proportion of diarrheal cases in children under five due to rotavirus declined from 38% before vaccine introduction to 23% after, a relative reduction of nearly 40% [5,12,26]. This shows that even a moderately effective vaccine can have a major public health impact when baseline disease burden is high.

**Table 1:** Summary of vaccine effectiveness studies for oral rotavirus vaccines in LMICs ^a,b^.

Vaccine	Country/Study Period	Age Group(s)	Vaccine Effectiveness (VE) % (95% CI)	Key Findings	Reference
Rotarix	Afghanistan; 4 centers; Study period: 2018–2021	6–59 months	45% (22–62)	Effectiveness remained stable despite political instability and health system transitions.	Anwari et al., 2024 [27]
Rotarix	Mozambique; 7 centers; Study period: 2017–2019	6–59 months	35% (−30–66)	VE appeared lower in stunted children, 14% (95% CI, −138 to 66), than in non-stunted children, 59% (95% CI, −125 to 91), although confidence intervals were wide and overlapping.	Chissaque et al., 2022 [28]
Rotarix	Kenya; 3 centers; Study period: July 2014–December 2017	<5 years	64% (35–80)	Robust protection was observed in children even beyond infancy with malnutrition having a detrimental effect on VE.	Khagayi et al., 2020 [29]
Rotarix	Uzbekistan; 2 centers; Study period: January 2014–December 2017	<5 years	51% (2–75)	VE point estimates were higher for more severe outcomes (55% for cases requiring intravenous fluids, 57% for hospitalization lasting at least 5 days, and 67% for cases with severity score ≥ 11)	Eraliev et al., 2021 [30]
Rotarix	Zimbabwe; 2 sites; Study period: March 2014–December 2017	6 months to <5 years	61% (21–81)	VE became non-significant in children aged ≥12 months, potentially due to waning immunity or naturally acquired immunity.	Mujuru et al., 2019 [31]
Rotarix	Bangladesh; Open-labelled cluster-randomized trial; Study period: November 2008–March 2011	<2 years	41.4% (23.2–55.2)	ORV vaccination significantly reduced rotavirus-associated gastroenteritis with overall VE being higher during the first year of life.	Zaman et al., 2017 [32]
Rotarix	Tanzania (Zanzibar); Single center; Study period: 2010–2015	5–23 months	57% (14–78)	The implementation of ORV had substantially reduced rotavirus-related hospitalizations prompted by high vaccine coverage.	Abeid et al., 2017 [22]
Rotarix	Malawi; Single center; Study period: 2012–2015	<5 years	58.3% (20.2–78.2)	Extended follow-up showed sustained population-level reductions in rotavirus-associated hospitalisations over multiple years.	Bar-Zeev et al., 2016 ^c^ [33]
Rotarix	Armenia; 2 centers; Study period: 2012–2015	6–23 months	62% (36–77)	High-severity disease protection was 79%. There was significant reduction in rotavirus hospitalisations among unvaccinated older children indicating substantial indirect benefits.	Sahakyan et al., 2016 [21]
Rotarix	Bolivia; 5 sites; Study period: 2013–2014	<5 years	59% (37–73)	Evaluation 5 years post-introduction showed that while VE against severe disease waned in children ≥1 year, effectiveness against “very severe” disease remained high.	Pringle et al., 2016 [20]
Rotarix	Botswana; 4 centers; Study period: June 2013–April 2015	<5 years	54% (23–73)	VE was 75% in children with no undernutrition but dropped to −28% (non-significant) in those with moderate or severe undernutrition.	Gastañaduy et al., 2016 [19]
Rotarix	Moldova; 2 centers; Study period: September 2009–July 2014	<5 years	79% (62–88)	The study found evidence of indirect protection, as rotavirus hospitalizations declined among unvaccinated children, consistent with a herd immunity effect.	Gheorghita et al., 2016 [34]
Rotarix	Ghana; 7 centers; Study period: April 2010–December 2014	6–23 months	60% (−2–84)	Significant reduction in pediatric diarrhoea disease after 3 years of rotavirus vaccine introduction with limited statistical precision of VE estimates.	Armah et al., 2016 [35]
Rotarix	Malawi; Single center; Study period: January 2012–June 2014	<5 years	64% (24–83)	Routine implementation of ORV showed significant reduction in hospitalizations especially in infants less than 1 year of age.	Bar-Zeev et al., 2015 ^c^ [36]
Rotarix	South Africa; 7 centers; Study period: April 2010–Oct 2012	18 weeks to 23 months	57% (40–68)	Conducted in a high HIV-prevalence setting, the study found similar VE in HIV-exposed-uninfected (HEU) (64%) and HIV-unexposed-uninfected children (HUU) (54%).	Groome et al., 2014 [18]
Rotarix	Colombia; 7 centers; Study period: 2011–January 2013	6 months to <5 years	84.42% (22.68–96.86)	Analysis indicated significant protection against overnight hospitalisation in children 6–11 months, but the lower VE observed in children >12 months needs to be evaluated further.	Cotes-Cantillo et al., 2014 [37]
Rotarix	Bolivia; 6 sites centers; Study period: March 2010–June 2011	<3 years	69% (54–79)	Monovalent rotavirus vaccine demonstrated significant protection against rotavirus diarrhoea with protection persisting until 2 years of life.	Patel et al., 2013 [17]
RotaTeq	Rwanda; 8 centers; Study period: September 2012–May 2015	6 months and older	80% (28–94)	A full series of vaccinations in the slightly delayed age cohort (7–18 weeks of age) demonstrated substantial protection against rotavirus hospitalizations and sustained protection beyond 1 year of life.	Tate et al., 2016 [23]
RotaTeq & Rotarix	Guatemala; 4 centers; January 2012–August 2013	6 months to <5 years	52% (26–69)	Concurrent use of RV1 and RV5 showed no significant difference in effectiveness between products; both were effective against the emerging G12P strain	Gastañaduy et al., 2016 [16]
Rotavac	India; 31 centers; Study period: January 2016–January 2020	6–59 months	54% (45–62)	The indigenous monovalent vaccine provided significant heterologous protection against non-vaccine strains G1P [8] and G3P [8] under routine programmatic use.	Nair et al., 2025 [38]
Rotasiil	India; 32 sites; Study period: July 2019–December 2023	4–23 months	52% (32–66)	Demonstrated real-world effectiveness under routine programmatic conditions, with strongest protection in early life and broad heterotypic strain coverage	Mohan et al., 2025 [39]
Rotasiil	DR Congo; two centers; Study period: 1 January 2020–December 2023)	6–30 months	60% (20–80)	Rotasiil demonstrated significant reductions in rotavirus-associated hospitalizations.	Lungayo et al., 2025 [40]

^a^ Detailed age, strain, and setting-specific effectiveness estimates from individual sentinel sites have been reported extensively elsewhere and are therefore not reproduced here. ^b^ Only primary post-licensure observational studies reporting individual-level vaccine effectiveness were considered for this table. Systematic reviews, meta-analyses, modelling studies, and effectiveness projections are not listed in this table. ^c^ Both studies originate from the same national surveillance system; the 2015 manuscript reports early post-introduction individual-level effectiveness, while the 2016 manuscript evaluates longer-term population-level impact and durability.

## 3. Effect of Malnutrition and HIV on ORV Performance

The causes of impaired ORV effectiveness are multifaceted and cannot be attributed to a single cause. The common barriers that affect ORV performance include interference by transplacental maternal antibodies, secretory IgA from breast milk, infant gut microbiome, environmental enteric dysfunction (EED), co-existence of other enteric pathogens, concurrent OPV administration, histo-blood group antigens (HBGAs) and other genetic factors which are discussed extensively in existing literature [14,15,41,42,43,44]. However, malnutrition and HIV are specifically discussed here because of their public health relevance in many LMIC settings and established effects on immune function. Malnutrition is often hypothesized as a primary cause of impaired ORV efficiency in LMICs due to its effect on gut integrity and mucosal immune response. However, clinical and field studies have produced inconsistent findings, with several analyses failing to demonstrate a clear and independent association between nutritional status and ORV immunogenicity [14,41,42,45,46]. This suggests that while malnutrition may contribute to a broader context of vulnerability, it is unlikely to act as a readily modifiable determinant of ORV underperformance in LMICs. Various studies done in African settings show that the HIV status of the infant did not have a profound impact on the immunogenicity of ORVs and is safe to be administered to infants living with HIV (iLHIV). Studies from Southern African countries indicate that HIV exposed uninfected (HEU) infants maintain significant protection against rotavirus disease in comparison with HIV unexposed uninfected (HUU) infants [33,41,47,48,49,50]. Also, it has been suggested that HEU infants demonstrate more robust immune response against the first dose owing to the lower levels of interference by transplacental maternal antibodies [18]. On the other hand, some studies have reported an attenuated protective effect in iLHIV cohorts. But these studies were underpowered to definitively quantify the VE in iLHIV due to the lack of statistical power and relevant control groups, thereby failing to yield a definitive conclusion [41,47]. Overall, the current evidence suggests that the performance of ORV is context-dependent and remains incompletely defined.

## 4. Can Oral Vaccines Perform Better in LMICs?

The underperformance of ORVs in LMICs has prompted the need to identify various strategies to optimize vaccine performance beyond that achieved with current standard infant schedules. Proposed approaches include modified dosing schedules, booster doses, heterologous vaccine regimens, and adjunct interventions. Currently, Rotarix is administered as a two-dose schedule whereas other WHO-prequalified vaccines follow a three-dose schedule, typically given at 4-week intervals starting at 6 weeks of age [5].

## 5. Alternate Dosing Schedules

Several clinical trials have assessed whether changing the timing or number of ORV doses can improve immunogenicity (Table 2). Studies from Malawi and South Africa, evaluating an additional Rotarix dose administered at 6 weeks (6, 10 & 14 weeks vs. 10 & 14 weeks) reported either no clear improvement or only small, non-significant gains in seroconversion [51,52]. Similarly, studies assessing an additional Rotarix dose at 14 weeks (administered at 6, 10 & 14 weeks vs. 6 & 10 weeks) produced inconsistent findings [53,54]. Overall, these findings suggest that extending the primary schedule may offer limited immunogenic benefit, although the effect is inconsistent across settings. Delayed primary dosing has also been explored to reduce interference from maternal antibodies and improve immune response. However, studies evaluating schedules at 10 and 14 weeks showed only modest and statistically non-significant improvements compared with the standard 6- and 10-week schedule [53,54]. In addition, delayed dosing raises important concerns, including reduced protection during a period of high risk, missed or incomplete vaccination, operational difficulties, and possible age-related safety concerns such as intussusception. For these reasons, early vaccination remains the preferred strategy [5,53].

While most studies evaluating the administration of an ORV booster dose, typically at around 9 months or alongside routine childhood vaccines, show that it significantly enhances immunogenicity, discordant results from Zambia suggest that effectiveness may be modulated by setting-specific factors [55,56,57]. Although these studies were not powered to detect rare adverse events, no cases of intussusception were reported. Similar findings have also been reported among the Aboriginal and Torres Strait Islander children in Australia, a high-risk population within the high-income setting that continues to experience disproportionately high burden of rotavirus disease despite routine rotavirus vaccination [58]. These findings suggest that booster doses may improve immune response. This conclusion is further supported by the systematic review by Middleton et al., which proposed that improved responses in late infancy may partly reflect declining maternal antibody levels after 6 months of age, although the exact mechanism remains uncertain [59]. However, important limitations remain. With the exception of one study [51], rest all focused on short-term immunogenicity rather than robust clinical outcomes. The serum anti-rotavirus IgA assessed in these studies remains an imperfect marker of protection, particularly in high-burden settings where it is difficult to differentiate between natural infections and vaccine responses [60]. In addition, randomized controlled data on booster doses given in the second year of life are still lacking, despite modelling studies suggesting that this approach could reduce rotavirus-related morbidity and mortality [61,62]. At present, these strategies should be viewed as incremental optimization measures rather than definitive solutions to the persistent effectiveness gap in high-burden settings.

**Table 2:** Studies on alternate dosing schedules of oral rotavirus vaccines.

Vaccine	Country	Study Period	Study Design	Strategy and Schedule	Key Findings	Reference
Rotarix	South Africa	September 2003–February 2004	Randomized, double-blinded, placebo-controlled trial	Additional primary-series dose at 6th week (i.e., 10 and 14 weeks vs. 6, 10 and 14 weeks)	Anti-rotavirus IgA seroconversion rates were similar in both 2-dose (44.3%) and 3-dose (44.4%) regimens	Steele et al., 2010 [51]
Rotarix	South Africa, Malawi	October 2005–January 2006, October 2006–July 2007	Double-blind, randomized, placebo- controlled multicenter study	Additional primary-series dose at 6th week (i.e., 10 and 14 weeks vs. 6, 10 and 14 weeks)	The additional dose regimen in South Africa showed a modest non-significant anti-rotavirus IgA seroconversion. In Malawi, there is no extra benefit of the additional dose. However, this finding may be underestimated due to higher incidence of natural rotavirus infections	Madhi et al., 2010 * [52]
Rotarix	Pakistan	April 2011–September 2012	Phase IV, open-label, randomized trial	Additional primary-series dose at 14th week (6 and 10 weeks vs. 6, 10 and 14 weeks)	No significant difference in seroconversion neither in the additional dose nor the delayed dose regimens	Ali et al., 2014 [53]
				Delayed primary schedule 6 and 10 weeks vs. 10 and 14 weeks		
Rotarix	Ghana	September 2012–February 2013	Phase 4, single center, individually randomized, open-label trial	Additional primary-series dose at 14th week (6 and 10 weeks vs. 6, 10 and 14 weeks)	Anti-rotavirus IgA seroconversion rate increased by 14.5% in individuals who received three doses of vaccine	Armah et al., 2016 [54]
				Delayed primary schedule (6 and 10 weeks vs. 10 and 14 weeks)	No significant difference in seroconversion	
Rotarix	Bangladesh	Study period: January–September 2013	Single-center, open-label, randomized, parallel-group trial	Booster dose at 9th month	The booster dose significantly increased anti-rotavirus serum antibody titres. IgA seropositivity rose from 52.7% to 69.6%, and IgG seropositivity increased from 66.3% to 88.3%	Zaman et al., 2016 [55]
				Co-administration with MR vaccine	There was no interference with the seropositivity of MR vaccine when the booster dose of rotavirus vaccine was administered concomitantly	
Rotarix	Zambia	September 2018–November 2018	Single-center, open-label, randomised, controlled trial	Booster dose at 9th month. 6 and 10 weeks vs. 6th week, 10th weeks, and 9th month	Good toleration of booster dose but no significant difference in geometric mean titres and ratios of anti-rotavirus IgA between the control and trial arms.	Laban et al., 2023 [57]
Rotarix	Australia	March 2018–August 2020	Phase IV, double-blind, randomized, placebo-controlled clinical trial	Booster dose in late infancy. 2 months and 4 months vs. 2 months, 4 months and 6–11 months	Anti-rotavirus IgA Seroconversion rate increased by 16% in individuals who received three doses of vaccine	Middleton et al., 2022 [58]
RotaTeq	Mali	October 2014–December 2014	Open-label, individual-randomized, comparative immunogenicity trial	Booster dose at 9th month	The anti-rotavirus IgA seropositivity in the booster dose group was 56.9% compared to 31.5% in the control group	Haidara et al., 2018 [56]
				Co-administration with Measles, Yellow fever and Meningitis A vaccines	Co-administration of a booster dose did not interfere with the immune responses to measles or meningitis A vaccines. But its interference with the yellow fever vaccine cannot be ruled out.	

* This multicentric randomised control trial generated two more secondary publications which are not included here to avoid double counting of evidence. MR—Measles and Rubella.

## 6. Interchangeability of Rotavirus Vaccine Products and Real-World Programme Switching

Evidence from studies evaluating specific mixed vaccine regimens suggests that heterologous regimens offer a practical way to maintain coverage and ensure programme continuity when completing a series with a single product is disrupted by supply, procurement, or population movement challenges in LMICs. The main purpose is to ensure that children complete a vaccination series despite operational constraints, rather than to improve vaccine potency. The first randomized trial, conducted in the United States, found that mixed regimens using RotaTeq and Rotarix were non-inferior in immunogenicity and had similar safety profiles compared to standard homologous schedules [63]. These findings were later supported by studies from India and Mexico as well [64,65] (Table 3). Beyond clinical trials, several countries have also changed ORV products within national immunization programmes because of supply shortages, market exits, or procurement changes. Evidence from Ghana and Palestine suggests that high vaccine coverage can be maintained during these transitions between oral rotavirus vaccine products [66,67,68]. In Palestine, reductions in diarrheal disease were maintained after the switch from Rotarix to Rotavac, suggesting continued public health benefit [66]. In Ghana, modelling analyses suggested that switching from the two-dose Rotarix schedule to the three-dose Rotavac schedule could increase overall VE, possibly because the third dose provides an additional opportunity for seroconversion [68]. In contrast, a temporary rise in mortality and rotavirus detection observed during the transition period in Zambia appears to have been driven by stock-outs and supply-chain disruption rather than by problems related to vaccine interchangeability itself [69]. Together, the available evidence indicates that heterologous regimens are immunologically non-inferior, comparably safe and programmatically useful when vaccine supply is unstable. These findings are reassuring for countries that must navigate procurement changes, market exits, or financial constraints while maintaining routine immunization services.

## 7. Other Interventions to Improve the Vaccine Performance

Withholding breastfeeding around the time of vaccination was studied to reduce possible interference from maternal antibodies and antiviral factors in breast milk, but trials from South Africa, India, and Pakistan showed no meaningful improvement in vaccine immune response [70,71,72]. Zinc and other micronutrient supplements were evaluated because of their role in mucosal immunity and intestinal health, but randomized studies found inconsistent or largely null effects on ORV immunogenicity, suggesting that isolated nutrient supplementation cannot overcome the complex nutritional deficits present in high-burden settings [42,45,73]. Microbiome modulation, including probiotic supplementation, has been evaluated in pediatric populations which have shown only modest and inconsistent benefits without clear improvement in clinical protection [45]. On the contrary, a proof-of-concept study evaluating antibiotic-mediated microbiome modulation in adults has provided mechanistic insights into ORV immunogenicity which may lay the foundation for future research [74]. Overall, these findings indicate that adjunct interventions have limited value as practical tools to improve ORV performance and are more useful for understanding the biological barriers that restrict VE in LMICs.

## 8. Next Generation Rotavirus Vaccines

Next-generation rotavirus vaccines are being developed to overcome the biological and programmatic limitations of current ORVs in LMICs and include improved live oral vaccines, parenteral non-replicating vaccines, and newer formulations designed to simplify delivery and improve programme fit.

**Novel Oral Rotavirus Vaccines:** Neonatal oral vaccines, given in the first days of life rather than at 6–8 weeks, may improve vaccine take because conditions in the early infant gut are more favourable, with lower gastric acidity, less established environmental enteric dysfunction, and fewer competing enteric infections [75,76,77]. The leading candidate, RV3-BB, is a naturally attenuated neonatal strain that can replicate in the infant gut even in the presence of maternal antibodies [76,77,78]. Phase 2B trial data from Indonesia demonstrated approximately 75% efficacy against severe rotavirus gastroenteritis, with a further trial in Malawi showing good immunogenicity and tolerability in a high-burden African setting [79,80]. Neonatal dosing may also reduce concerns about intussusception because the vaccine is given at an age when the natural risk is very low [76]. Regionally developed oral vaccines, such as Rotavin-M1 in Vietnam and newer multivalent candidates in China, also from a programmatic perspective, indigenous vaccine products may improve domestic supply and affordability, although evidence from large multi-country studies remains limited [76,77,81,82].

**Parenteral vaccines:** Injectable and non-replicating vaccines aim to bypass gut-level barriers such as maternal antibodies, enteric infections, and intestinal inflammation by inducing systemic immunity rather than depending on replication in the gut. In addition to overcoming biological constraints, injectable rotavirus vaccines further facilitate integration with existing childhood immunisation schedules through co-administration or future combination with other injectable paediatric vaccines, potentially improving programmatic efficiency and reducing logistical complexity. Candidates such as the VP8 subunit vaccine have shown promising safety and immunogenicity in early trials, but important questions remain about long-term protection, correlates of immunity, and real-world effectiveness [75,78,83,84]. Other platforms, including inactivated whole-virus, mRNA, and virus-like particle vaccines, are still in early stages of development [75,76,78].

**Formulations and Delivery Innovations:** Formulation advances are also important for improving rotavirus vaccine use in LMICs. Newer products are being designed to reduce cold-chain dependence, simplify administration, and improve thermostability [75,76]. Examples include liquid and heat-stable formulations such as ROTAVAC 5D and Rotasiil, which may reduce wastage and make vaccine delivery easier in resource-limited settings [78,83,85,86,87]. Furthermore, the Rotavin-M1 product also transitioned from a frozen precursor to non-frozen liquid presentation with similar immunogenic results as that of the former product [81]. These innovations may not directly improve biological efficacy, but they can strengthen programme sustainability, supply reliability, and coverage.

## 9. Financing, Procurement, and Long-Term Sustainability of Rotavirus Vaccination

In LMICs, early introduction and continued use of ORVs are shaped by more than VE alone. Vaccine price, financing, supply security, and programme feasibility are also major determinants of policy decisions [88,89]. Gavi has played a major role in supporting early access and reducing financial risk, but the transition to full domestic financing creates important affordability challenges, especially when countries lose access to Gavi-negotiated prices [88,89,90,91,92]. Despite this, rotavirus vaccination remains a high-value public health investment because it reduces hospitalizations, severe disease, and deaths, often offsetting a substantial share of programme costs. Evidence from countries such as Bangladesh, Ghana, and Malawi suggests that post-transition difficulties are driven more by budget and procurement constraints than by poor vaccine value [90,93]. Thus, long-term programme sustainability depends increasingly on affordability and supply stability, supported by strategies such as market diversification and pooled procurement [90,93,94].

The availability of multiple WHO-prequalified vaccines gives countries more flexibility in procurement and reduces reliance on a single manufacturer. This allows national programmes to choose products based on price, supply, and logistical needs. However, diversification can also increase programme complexity, because lower-cost products may differ in dosing schedule, storage requirements, presentation, and wastage [88,90,92,94,95,96,97]. Thus, while market diversification can strengthen supply resilience, it also increases the need for strong procurement planning and operational capacity.

Within this diversified landscape, pooled procurement has emerged as a key mechanism for improving sustainable access [88]. By combining demand through platforms such as UNICEF procurement or the Pan American Health Organization (PAHO) revolving fund, pooled purchasing enables price negotiation, increases supply predictability and reduces market risk for individual countries [88,94]. This approach is particularly critical following Gavi transition, when countries face rising financial responsibilities and potential loss of preferential pricing. In conclusion, economic constraints exert an indirect but profound influence on vaccine equity and continuity of coverage. As countries move toward self-financing, programme stability depends not only on vaccine price, but also on how well financing, procurement, and delivery systems work together.

## 10. Conclusions

Rotavirus vaccination remains the most effective strategy to reduce disease burden in LMICs, despite biological and programmatic challenges. Evidence shows that long-term impact depends not only on vaccine performance but also on how and where the vaccine is implemented. The key question for policymakers is no longer whether to vaccinate, but how to prioritize efforts to sustain and optimize vaccine impact in changing contexts. This requires a balanced approach across three areas: strengthening early infant vaccination and surveillance to maximize current vaccine benefits; ensuring economic sustainability through strategies such as diversified supply and efficient procurement; and supporting the development of next-generation vaccines to address existing limitations. Ultimately, reducing rotavirus burden in LMICs will depend on increasing coverage to meet set targets, adapting to local health system realities, and integrating scientific advances with practical program delivery.

## Figures and Tables

**Table 3:** Mixed oral rotavirus vaccine regimen trials.

Setting	USA	India	Mexico
Study design	Randomized, non-blinded, multicenter study	Multicentre, open-label, randomised, controlled, phase 4, non-inferiority trial	Randomized, double-blinded study
Randomized cohorts	5 groups * Group 1—RV1 + RV1 * Group 2—RV5 + RV5 + RV5; Group 3—RV1 + RV5 + RV5; Group 4—RV5 + RV1 + RV1; and Group 5—RV5 + RV5 + RV1;	6 groups * Group1: Rotavac–Rotavac–Rotavac * Group2: Rotasiil–Rotasiil–Rotasiil Group3: Rotavac–Rotasiil–Rotavac Group4: Rotasiil–Rotavac–Rotasiil Group5: Rotavac–Rotasiil–Rotasiil and Group6: Rotasiil–Rotavac–Rotavac	7 groups * Group 1—RV1 + RV1 + placebo; * Group 2—RV5 + RV5 + RV5; Group 3—RV1 + RV5 + RV5; Group 4—RV5 + RV1 + RV1; Group 5—RV5 + RV5 + RV1; Group 6—RV5 + RV1 + RV5; and Group 7—RV1 + RV5 + RV1
Key findings	All these trials found that the administration of oral rotavirus vaccines interchangeably was immunologically non-inferior with comparable seroresponse rates and no significant safety concerns. However, the findings were limited to the short-term immunologic outcomes and the clinical impact of these findings was not evaluated.		
Reference	Libster et al., 2016 [63]	Kanungo et al., 2022 [64]	Macías-Parra et al., 2024 [65]

* refers to the standard vaccine regimens. RV1—Rotarix; RV5—Rotateq.

## Data Availability

No new data were created or analyzed in this study. Data sharing is not applicable to this article.
